# Biogeographical and biodiversity patterns of planktonic microeukaryotes along the tropical western to eastern Pacific Ocean transect revealed by metabarcoding

**DOI:** 10.1128/spectrum.02424-23

**Published:** 2024-03-15

**Authors:** Yingjun Fu, Zhishuai Qu, Ying Wang, Ping Sun, Nianzhi Jiao, Dapeng Xu

**Affiliations:** 1 State Key Laboratory of Marine Environmental Science, College of Ocean and Earth Sciences, Institute of Marine Microbes and Ecospheres, Xiamen University, Xiamen, China; 2 Fujian Key Laboratory of Marine Carbon Sequestration, Xiamen University, Xiamen, China; 3 Institute of Marine Science and Technology, Shandong University, Qingdao 266237, China; 4 Key Laboratory of Ministry of Education for Coastal and Wetland Ecosystems, Fujian Provincial Key Laboratory of Coastal Ecology and Environmental Studies, College of the Environment and Ecology, Xiamen University, Xiamen, China; Institut Ruder Boskovic, Zagreb, Croatia

**Keywords:** microbial eukaryotes, protists, Syndiniales, SSU rRNA gene, high throughput sequencing

## Abstract

Microeukaryotic plankton (0.2–200 µm), which are morphologically and genetically highly diverse, play a crucial role in ocean productivity and carbon consumption. The Pacific Ocean (PO), one of the world’s largest oligotrophic regions, remains largely unexplored in terms of the biogeography and biodiversity of microeukaryotes based on large-scale sampling. We investigated the horizontal distribution of microeukaryotes along a 16,000 km transect from the west to the east of the PO. The alpha diversity indices showed a distinct decreasing trend from west to east, which was highly correlated with water temperature. The microeukaryotic community, which was clustered into the western, central, and eastern PO groups, displayed a significant distance-decay relationship. Syndiniales, a lineage of parasitic dinoflagellates, was ubiquitously distributed along the transect and dominated the community in terms of both sequence and zero-radius operational taxonomic unit (ZOTU) proportions. The prevailing dominance of Syndiniales-affiliated ZOTUs and their close associations with dinoflagellates, diatoms, and radiolarians, as revealed by SparCC correlation analysis, suggested that parasitism may be an important trophic strategy in the surface waters of the PO. Geographical distance and temperature were the most important environmental factors that significantly correlated with community structure. Overall, our study sheds more light on the distribution pattern of both alpha and beta diversities of microeukaryotic communities and highlighted the importance of parasitisms by Syndiniales across the tropical PO.

**IMPORTANCE:**

Understanding the biogeographical and biodiversity patterns of microeukaryotic communities is essential to comprehending their roles in biogeochemical cycling. In this study, planktonic microeukaryotes were collected along a west-to-east Pacific Ocean transect (ca. 16,000 km). Our study revealed that the alpha diversity indices were highly correlated with water temperature, and the microeukaryotic communities displayed a distinct geographical distance-driven pattern. The predominance of the parasitic dinoflagellate lineage Syndiniales and their close relationship with other microeukaryotic groups suggest that parasitism may be a crucial survival strategy for microeukaryotes in the surface waters of the Pacific Ocean. Our findings expand our understanding of the biodiversity and biogeographical pattern of microeukaryotes and highlight the significance of parasitic Syndiniales in the surface ocean.

## INTRODUCTION

Microeukaryotic plankton (0.2–200 µm in size, including protists, fungi, and small zooplankton) are widely recognized as significant contributors to marine productivity and carbon consumption (1–4). Due to their remarkable morphological and genetic diversity, microeukaryotes are present in virtually all marine habitats and serve multiple and essential roles in the biogeochemical cycling of the world’s oceans (5). Photosynthetic microeukaryotes, including diatoms and dinoflagellates, constitute the base of the ocean food webs as major primary producers in the euphotic zone (6). Higher trophic level microeukaryotes, such as ciliates and diverse flagellates, which consume picophytoplankton and bacteria, are preyed upon by larger zooplankton (6–8). Mixotrophic microeukaryotes, which are capable of performing both phototrophy and phagotrophy, can increase trophic transfer to higher levels in the marine food web, enhance the efficiency of the biological carbon pump, and increase carbon sequestration in the deep ocean (9). These intricate behavioral strategies and organismal interactions have complicated the construction of a comprehensive model of the carbon cycle (6).

The surface layer of the world’s oceans serves as a biogeochemical membrane separating the atmosphere and the interior of the ocean (10). Over the past two decades, advances in high-throughput sequencing have enabled the examination of microeukaryotic biodiversity and the investigation of their ecological significance in marine environments (10). Previous research has established a significant correlation between marine planktonic microeukaryotes and abiotic factors, including spatial factors, temperature, salinity, chlorophyll (Chl) *a*, and nutrients (11–13). Recent evidence also indicates that biotic factors, such as prey availability, top-down grazing, and various ecological processes, such as dispersal limitation and environmental stresses, also influence microeukaryotic community structure (14–18). Furthermore, a deeper understanding of the co-occurrence patterns of microeukaryotes may contribute to the advancement of knowledge regarding the interactions between microbes in diverse natural marine environments (17–20).

Recent years have seen the advances of biodiversity studies on microeukaryotes in the Pacific Ocean (PO), the largest marine habitat on Earth, by applying sequencing-based techniques. Studies have shown a significant distance-decay relationship on the horizontal scaling of microeukaryotic communities (21), the different ecological processes governing the assembly processes of microeukaryotic communities in different depth zones (18), the temporal dynamics of microeukaryotic communities and the environmental driving factors (22–24), and the spatial distribution pattern and metabolic activities of microeukaryotic communities (4, 10, 25–28). However, previous studies were either based on a local scale sampling of a relatively small number of stations, focused on the vertical distribution of microeukaryotic communities, integrated data from multiple independent cruises, targeted only specific microeukaryotic groups, or examined the temporal dynamics of microeukaryotes at a fixed station. To our knowledge, no attempts have ever been made to infer the distribution pattern of microeukaryotes along the west-to-east transect across the tropical PO. More research needs to be done on the diversity distribution, community composition, community assembly process, and environmental driving factors of planktonic microeukaryotes in the PO.

By utilizing high-throughput sequencing on the V9 hyper-variable regions of the SSU rRNA gene, we surveyed microeukaryotes spanning 16,000 km of the PO in this study. The objectives of this study were to (i) disclose the spatial distribution patterns of the biodiversity, composition, and community assembly processes of major microeukaryotic groups and (ii) identify the abiotic and biotic factors that shape microeukaryotic communities.

## MATERIALS AND METHODS

### Sample collection

**Fig 1:**
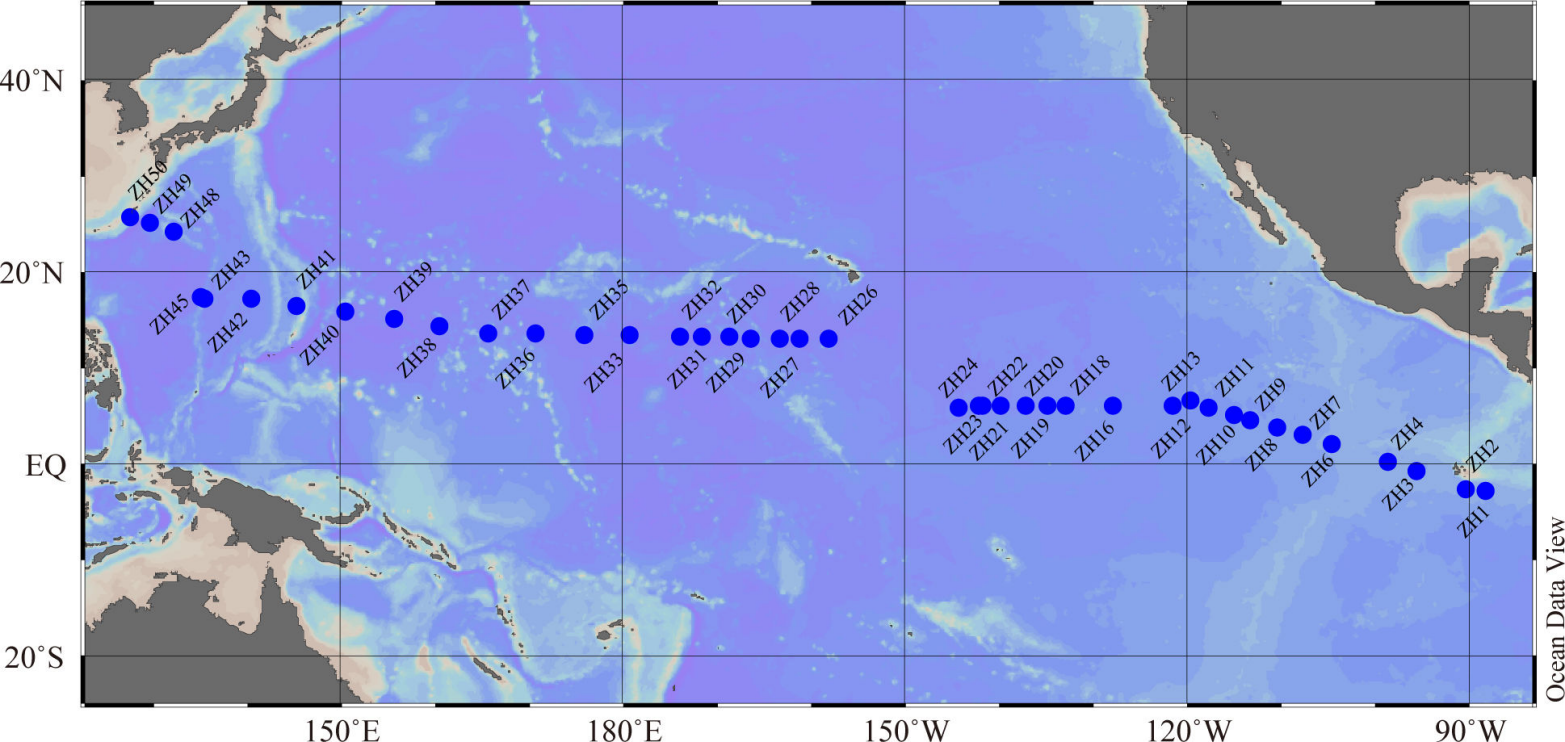
Location of the sampling sites.

Samples were collected onboard R/V Dayang No. 1 from 25 October to 11 December 2011 along a 16,000 km transect from the western to eastern PO (Fig. 1). In total, 41 samples of surface (0.5 m) seawater were collected. At each sampling station, 2 L of seawater was collected with Niskin bottles attached to a CTD rosette system. Seawater samples were pre-filtered through a 200-µm mesh (Nitex, Sefar), and the filtrates were collected on a membrane with a pore size of 0.22 µm and a 47-mm diameter (PALL, USA). The membranes were then flash frozen in liquid nitrogen and stored at −80°C for further DNA extraction. The environmental parameters, including sea water temperature, salinity, Chl *a* concentration, and abundance of *Prochlorococcus*, *Synechococcus*, pico-sized pigmented eukaryotes (PPEs), bacteria, and viruses [including high fluorescence virus (HFV) and low fluorescence virus (LFV)], were derived from Liang et al. (29) during the same cruise with the present study (29).

DNA was extracted using the PowerWater DNA Isolation Kit (MoBio Laboratories, Inc., Carlsbad, CA) according to Xu et al. (30). The obtained DNA was used to amplify the hypervariable V9 region of the SSU rRNA gene using the primer set 1389F/1510R (31). Each sample underwent three separate PCRs, which were then combined to obtain sufficient amplicons for sequencing. The Wizard SV Gel and PCR Clean-Up System (Promega, Beijing, China) was used to pool and purify PCR amplicons from the same sample. Paired-end (2 × 250 bp) multiplexed sequencing was performed by a commercial company using the Illumina MiSeq platform. All the sequences from this study have been deposited in the public NCBI Sequence Read Archive database under BioProject accession number PRJNA1060468.

### Sequence data processing

Trimmomatic (32) and Flash (33) were used to screen and assemble raw reads, and the employed criteria followed Li et al. (34) (34). The quality-filtered reads were then dereplicated using Usearch 11 (35). Reads were denoised (including the removal of potential chimeras) and clustered into biological zero-radius operational taxonomic units (ZOTUs) using UNOISE3 (36). The taxonomic assignment of the generated ZOTUs was achieved using SINTAX (37) by comparing them against the Protist Ribosomal Reference database (PR2, 38). The ZOTUs identified were classified into four trophic functional groups, i.e., phototrophs, heterotrophs, mixotrophs, parasites and not determined, according to references (39–41). Singletons and ZOTUs unaffiliated with Eukaryota were excluded from subsequent analyses.

### Statistical analyses

The alpha diversity indices, including ZOTU richness, Shannon, and Faith’s phylogenetic diversity (PD), were computed in Mothur (42) based on multiple random resampling at the lowest read counts (11,783) among all samples. Spearman’s correlation coefficients between alpha diversity indices and environmental variables were calculated using SPSS V.20.0 (43). To assess the combined effects of environmental variables on alpha diversity, multiple linear regression (MLR) models using the “lm” function in R were constructed (44). Before performing the MLR analysis, the logarithmic transformation of the data was performed, and the discrete points were excluded. We assessed the collinearity of the variables by calculating the variance inflation factor (VIF) using the “vif” function of the “car” package in R (45). The factors were included in the MLR analyses, only when the collinearity VIF <10. For selecting the optimal subset of variables in the final multiple regression models, we employed backward selection with the “stepAIC” function from the “MASS” package (46), and the insignificant variables were subsequently deleted. Finally, the relative importance of the variables for the final models was calculated using the “relweights” function (44).

The beta diversity was measured using Bray-Curtis distances and unweighted Unifrac distance, and the results were displayed by principal coordinate analysis (PCoA) with the “vegan” package in R (47). Using the same package, a similarity analysis (ANOSIM) was conducted to determine the significance of differences in community composition between the identified sample groups. To assess correlations between environmental variables and community variability, Mantel tests were conducted with the “vegan” package (48). With variance partitioning analysis (VPA) based on canonical correspondence analysis, the contributions of geographical distance, environmental, and biotic factors to the variances of microeukaryotic communities were analyzed. VPA was conducted using the “vegan” package (49).

To determine the relationships between Syndiniales and other microeukaryotic groups, correlation analyses were conducted using SparCC (50). To reduce the complexity of the network analysis, ZOTUs found in less than 1/3 of samples and with a relative abundance of less than 0.01% were excluded from the analysis. Following these thresholds, a total of 1,082 ZOTUs were run against one another using SparCC to identify significant correlations. The robust correlations were exported as a GML (Graph Modeling Language) format network file with correlation coefficient (*r*) values of ≥0.6 and false discovery rate-corrected *P* values of 0.01 (51). The network was visualized using Gephi v.0.9.2 (52).

## RESULTS

### The partitioning of microeukaryotic communities

**Fig 2:**
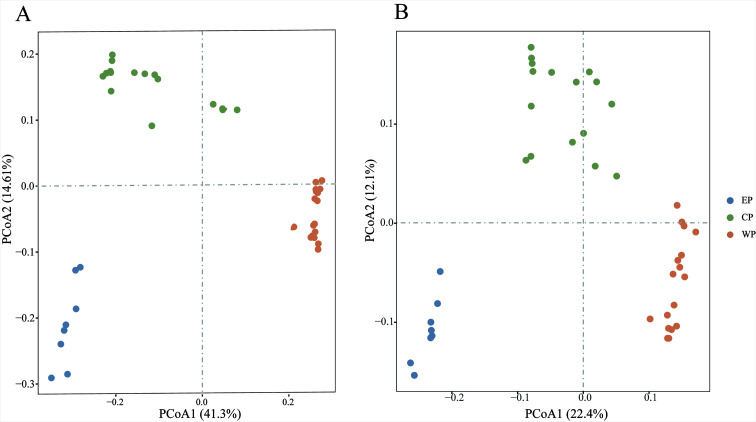
Principal coordinate analysis (PCoA) plots of microeukaryotic communities based on Bray-Curtis dissimilarities (**A**) and unweighted Unifrac distance (**B**).

Microeukaryotic communities were clustered into three regional groups based on Bray-Curtis dissimilarities and unweighted Unifrac distances (Fig. 2). These groups basically corresponded to their locations along the transect, i.e., the eastern PO group (EP, including ZH1–ZH9), the central PO group (CP, including ZH10–ZH29), and the western PO group (WP, including ZH30–ZH50). The unweighted pair group method with arithmetic mean (UPGMA) clustering dendrogram exhibited the identical pattern (Fig. S1). Statistically significant differences were identified among these groups using the ANOSIM (Table 1; *r* = 0.939, *P* < 0.001).

**TABLE 1:** Analysis of similarities of microeukaryotic communities among different groups^*a*^

	*R*	ρ
Global	0.939	0.0001
EP vs CP	0.850	0.0001
EP vs WP	0.893	0.0001
CP vs WP	0.924	0.0001

^*a*^ EP, the eastern Pacific Ocean group; CP, the central Pacific Ocean group; WP, the western Pacific Ocean group.

### Alpha diversity indices and microeukaryotic community composition

**Fig 3:**
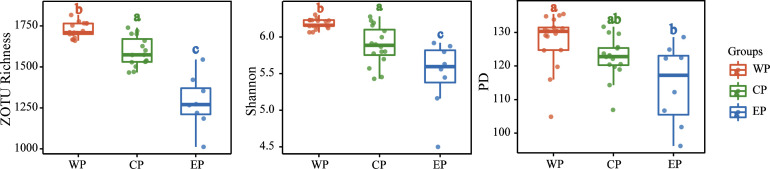
Comparison of alpha diversity indices (ZOTU richness, Shannon, and PD) among the three geographic groups as demonstrated by boxplots. The upper and lower boundaries of each box represent the 75th and 25th quartile values, respectively, while the lines within each box represent the median values. Bars without shared letters indicate significant differences at the level of *P* < 0.05 according to the Kruskal-Wallis test. WP, the western Pacific Ocean group; CP, the central Pacific Ocean group; EP, the eastern Pacific Ocean group.

After quality filtering and the removal of singletons and ZOTUs unaffiliated with Eukaryota, a total of 6,199 ZOTUs, ranging from 1,457 to 3,206 ZOTUs per sample, were obtained (Table S1). After being rarefied at the lowest sequence count (11,783, sample ZH48) among all samples, a total of 5,957 ZOTUs were recovered, ranging from 1,012 to 1,817 per sample (Table S1). All three indices demonstrated a statistically significant decreasing trend from the western PO to the eastern PO (*P* < 0.01; Fig. 3; Fig. S2). All three alpha diversity indices were significantly correlated with temperature and the abundances of *Synechococcus*, PPEs, and HFV, according to a Spearman correlation analysis between the alpha diversity indices and environmental variables (Table S2). The MLR model showed that water temperature was the most important environmental factor that correlated with the alpha diversity indices. It explained ca. 77%, 34%, and 58% of the variations in ZOTU richness, PD, and Shannon, respectively (Fig. S3).

Combining data from all sites gave an initial insight into the composition of the microeukaryotic community across the PO. Over half of the reads were affiliated with Alveolata (primarily Dinoflagellata and Ciliophora). Stramenopiles represented 11.7% of reads, followed by Opisthokonta, Hacrobia, Archaeplastida, and Rhizaria. The rest groups collectively contributed only 1.4% of all reads (Fig. S4A and C). In terms of ZOTU richness, Alveolata (mainly Dinoflagellata and Ciliophora) and Stramenopiles accounted for 67.6% and 13.1% of the total ZOTUs, respectively, being the top 2 contributors of ZOTU richness (Fig. S4B and D).

**Fig 4:**
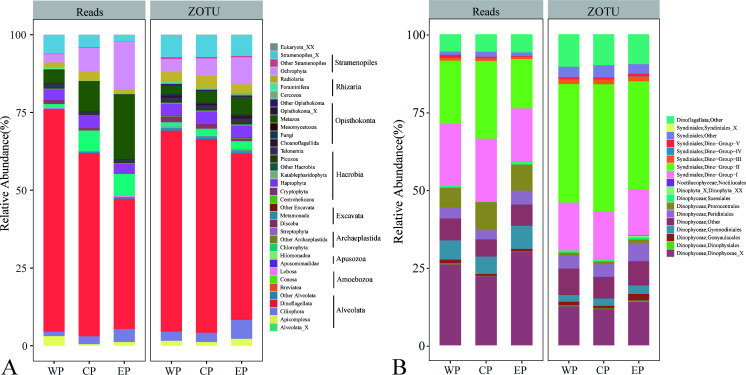
Proportions of reads and ZOTU richness of total microeukaryotes (**A**) and Dinoflagellata (**B**) in the three geographical groups (WP, CP, and EP). WP, the western Pacific Ocean group; CP, the central Pacific Ocean group; EP, the eastern Pacific Ocean group.

**Fig 5:**
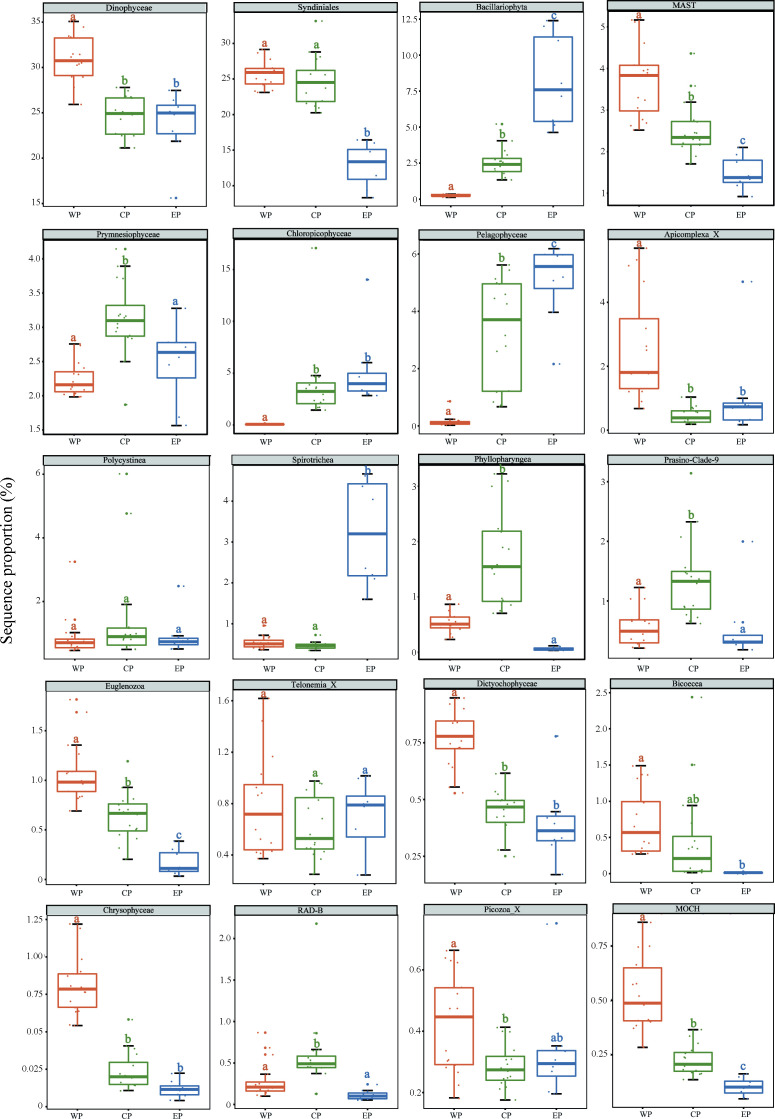
Box plots of the top 20 microeukaryote classes with the highest sequence proportions in three geographical groups (WP, CP, and EP). The upper and lower boundaries of each box represent the 75th and 25th quartile values, respectively, while the lines within each box represent the median values. Bars without shared letters indicate significant differences at the level of *P* < 0.05 according to the Kruskal-Wallis test.

Major microeukaryotic assemblages exhibited distinct spatial distribution patterns. Dinoflagellata dominated Alveolata, comprising ca. 60.8% of total reads (Fig. 4A; Fig. S4C). Notably, their proportion decreased from WP (ca. 70.71%) to EP (ca. 41.79%). Within Dinoflagellata, the sequence proportion of Syndiniales was higher in the WP than in the EP (Fig. 5). Dino-group-I and Dino-group-II dominated Syndiniales, with the latter accounting for 40% of the total Syndiniales ZOTUs (Fig. 4B). The sequence proportions of Bacillariophyta, Chloropicophyceae, Pelagophyceae, and Spirotrichea increased from WP to EP, whereas those of Dinophyceae, Syndiniales, MAST, Apicomplexa, Euglenozoa, Dictyochphyceae, Bicoecea, Chrysophyceae, Picozoa_X, and MOCH decreased (Fig. 5). The highest sequence proportions of Prymnesiophyceae, Phyllopharyngea, Prasino-Clade-9, and RAD-B were observed in CP, whereas those of Polycystinea and Telonemia_X did not differ significantly between WP, CP, and EP (Fig. 5).

The majority of the 150 ZOTUs found across all samples belonged to Dinoflagellata, with 18 belonging to Dino-Group-I, 16 to Dino-Group-II, and 1 to Dino-Group-III (Fig. S5). Only one ZOTU (ZOTU 3) was found to be abundant (representing >1% of sequences within a given sample) in all samples. ZOTU one was abundant in all samples except for ZH8. Several ZOTUs, including ZOTU 6, ZOTU 5, ZOTU 7, ZOTU 10, and ZOTU 13, were abundant in the majority of samples while intermediate (representing 0.01%–1% of sequences within a given sample) but never rare (representing <0.01% of sequences within a given sample). The remaining ZOTUs were intermediate in the majority of samples, never found to be abundant, and rare only in a few samples (Fig. S5).

To infer the possible interactions between Syndiniales and hosts, a co-occurrence network analysis was conducted using SparCC. After screening, 434 ZOTUs were left, which included members affiliated with Dino-Group-I (52), Dino-Group-II (67), Dino-Group-III (3), Dino-Group-V (3), and Syndiniales_X (3), representing ca. 29.5% of all ZOTUs (Fig. S6). There were 8,584 significant (*P* < 0.01) correlations with *r* ≥ 0.6, of which 3,753 involved a Syndiniales ZOTU (3,170 were related to non-Syndiniales taxa and 583 were among Syndiniales, respectively) (Table S3). Significant correlations among Syndiniales ZOTUs and with other ZOTUs included members belonging to dinoflagellates (72), diatoms (43), ciliates (16), chlorophytes (18), metazoans (33), MAST (17), haptophytes (11), and other groups (53).

### Distribution of the functional groups

Phototrophs were found to be more abundant (29.8% vs 22% of reads) but less diverse (18.2% vs 23.8% of ZOTU richness) than heterotrophs. The ratio of phototrophs to heterotrophs decreased from west to east (Fig. S7 and S8). The parasitic group exhibited greater abundance and diversity, comprising 26%–54% of reads and 64% of ZOTU richness. In addition, mixotrophic group was more prevalent in the WP, where its abundance and diversity occasionally surpassed those of metazoans (Fig. S7 and S8).

### Effects of environmental variables on the microeukaryotic communities

The Mantel test revealed a significant positive correlation (*r* = 0.71, *P* < 0.001, Table 2) between the community dissimilarity and geographic distance (i.e., the distance-decay pattern, Fig. S9). In addition, other variables, including temperature, salinity, Chl *a*, and the abundances of *Synechococcus*, PPEs, bacteria, and viruses, were significantly correlated with the dissimilarity of the communities.

**TABLE 2:** Mantel test for the correlation between microeukaryotic community and environmental factors^*a*^

	ρ	*R*
Geographical distance	**0.0001**	**0.711**
Temperature	**0.001**	**0.564**
Salinity	**0.001**	**0.308**
Chl *a*	**0.001**	**0.461**
*Prochlorococcus*	0.023	0.133
*Synechococcus*	**0.001**	**0.496**
PPE	**0.001**	**0.587**
Bacteria	**0.001**	**0.336**
HFV	**0.001**	**0.422**
LFV	**0.001**	**0.483**
Total viruses	**0.001**	**0.505**

^*a*^ The numbers in bold indicate statistically significant results.

**Fig 6:**
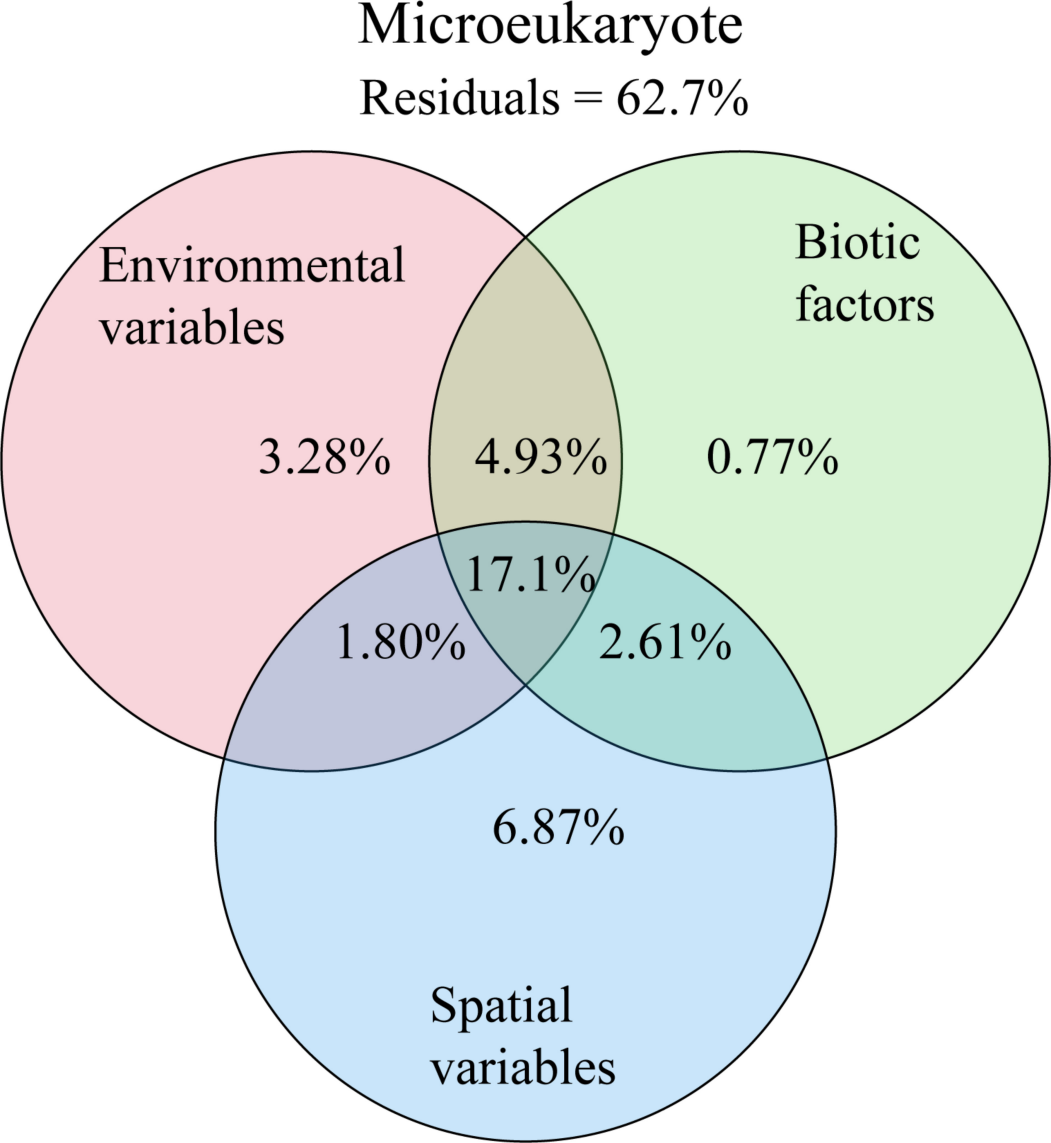
Variation partitioning analysis showing the effects of geographic distance, biotic factors, and environment on the community structure of microeukaryotes.

The VPA confirmed that geographic distance was the most influential factor on microeukaryotic communities, accounting for 6.87% of the variance. Environmental factors, including temperature, salinity, and Chl *a*, explained 3.28% of the variance, whereas biotic factors, including the abundance of *Prochlorococcus*, *Synechococcus*, bacteria, PPEs, and viruses, accounted for only 0.77% (Fig. 6). The residual 62.7% of the variance could not be explained by the model.

**Fig 7:**
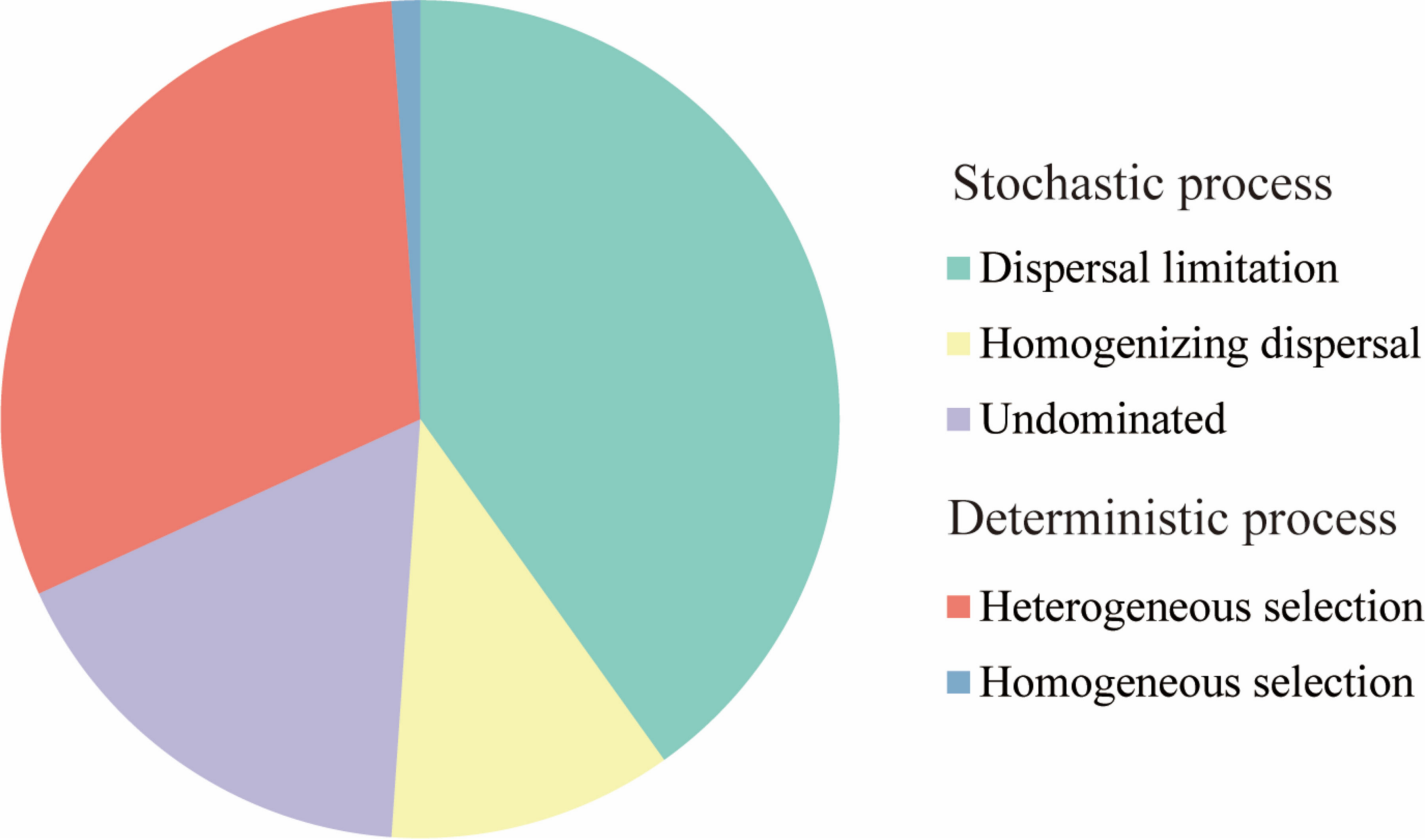
Partition of community assembly processes of microeukaryotes.

The phylogenetic null model analysis showed that dispersal limitation (40.1%) and heterogeneous selection (30.7%) were the primary drivers of microeukaryotic communities, followed by ecological drift (17.1%), homogenizing dispersal (10.9%), and homogeneous selection (1.1%) (Fig. 7).

## DISCUSSION

### Temperature as the main driver of the alpha diversity of microeukaryotes

In the present study, seawater temperature was positively and significantly correlated with all three alpha diversity indices of microeukaryotes along the 16,000 km PO transect. In fact, it has been widely reported that temperature is one of the most important factors influencing changes in the alpha diversity of terrestrial, freshwater, and marine organisms, especially on large spatial scales. For example, temperature was proposed to be the major factor responsible for the latitudinal diversity gradient (LDG) of organisms (41, 53, 54). For surface water planktonic microeukaryotes, the maximum alpha diversity was usually found in tropical to subtropical regions and then decreased toward the poles (53). The present study only encompassed a relatively limited latitudinal gradient, from the equatorial to 25 N, and alpha diversity indices increased rather than decreased from the equatorial to 25 N. The observed decreasing of seawater temperature from the equatorial (the eastern PO) to 25 N (the western PO) (Fig. S10) was one of the prominent atmosphere-ocean state features in the tropical PO, which is characterized by high sea surface temperature in the western Pacific warm pool and low sea surface temperature in the eastern Pacific tongue (55). Our findings appear to contradict the LDG. Nonetheless, the Spearman correlation analysis revealed a significant and positive correlation between seawater temperature and the alpha diversity indices of microeukaryotes. Furthermore, the MLR model analysis identified water temperature as the most important environmental factor that correlated with alpha diversity indices, which may serve as evidence that temperature is the primary driving factor responsible for the LDG.

### The prevalence of syndiniales across the PO

It has been reported that the dinoflagellate lineage Syndiniales is a major parasitic group of protists. Members of Syndiniales are parasites of dinoflagellates, ciliates, and even multicellular animals (56). In contrast to other parasitic organisms, Syndiniales parasites typically kill their hosts, thus being named as parasitoids (15). For example, it has been reported that certain members of the genus *Euduboscquella* can infect tintinnids and phagocytize part or the entire host cell (57). Overall, Parasitoids induced host mortality can rival grazing effects of larger zooplankton and promotes recycling of material within the microbial loop (58). The parasitic dinoflagellate lineage Syndiniales, which consists of Dino-Group-I, -II, -III, -IV, and -V (also known as marine alveolate, MALV I–V), was found to be the most abundant (sequence proportion) and diverse (ZOTU richness) microeukaryotic group in this study. In addition, 150 ZOTUs were found in all samples, of which 35 were affiliated with Syndiniales (18 with Dino-Group-I, 16 with Dino-Group-II, and 1 with Dino-Group-III, respectively). With the exception of a few samples, the majority of these Syndiniales ZOTUs were either abundant or intermediate in most samples, confirming their prevalence in the surface waters of the PO. A large proportion of Dinoflagellata sequences from various marine ecosystems have been reported to be represented by Syndiniales (19, 59, 60). Using data from the Tara Ocean global expedition, a study found that Syndiniales are highly abundant and ubiquitous in the world’s oceans (10). Based on weekly sampling at the Scripps Institution of Oceanography pier over the course of a year, Nagarkar and Palenik (61) determined that Syndiniales is the most species-rich and abundant taxonomic group (61). In the present study, based on the SparCC correlation analysis, after screening (see Materials and Methods for details), 434 ZOTUs with significant pairwise correlations were identified, 128 of which belonged to Syndiniales, accounting for ca. 29.5% of all ZOTUs. Of the 8,584 significant pairwise correlations discovered, 3,753 (43.7%) involved a Syndiniales ZOTU. The aforementioned results indicated that Syndiniales-affiliated ZOTUs were not only dominant but may also interact closely with microeukaryotic community members (62). Meanwhile, our study identified a wide variety of potential Syndiniales hosts (i.e., correlation-based interactions), including dinoflagellates, diatoms, ciliates, chlorophytes, MAST, metazoans, and haptophytes, which is consistent with previous studies based on laboratory cocultures and environmental surveys (19, 56, 61, 63–65). However, the correlations found between Syndiniales and certain microeukaryotic groups should not necessarily be interpreted as parasitisms. For example, it has been reported that the oligotrich ciliate *Strobilidium* sp. can rapidly consume the infective dinospores of *Amoebophrya* sp. (66). In a recent study that was based on the size fractionated sampling (i.e., comparing Syndiniales in the >0.2 µm and >10 µm fractions), Nagarkar and Palenik (61) proposed that the majority of the recovered Syndiniales sequences did not represent active infections but rather the free-living dinospore stage (61). In this study, the 0.2–200 µm fraction of the microeukaryotic community was collected. Therefore, we were unable to determine whether the Syndiniales recovered were free-living dinospores or whether they were in the infecting stages. Additional research employing size-fractionated sampling, RNA-based sequencing, and single-cell sequencing of Syndiniales and their potential hosts may shed more light on the stages, co-occurrence relationships, and potential roles of Syndiniales and their hosts in the biogeochemical cycling of the world’s oceans.

### Environmental influencing factors of microeukaryote beta diversities

In recent years, there has been a surge in research that investigates the diversity and distribution of microeukaryotes across diverse marine environments, including the PO (10, 18, 21, 41, 67). In our study, a discernible horizontal distribution pattern of planktonic microeukaryotic communities in surface water across the PO was identified. These microeukaryotic communities were clustered into the EP, CP, and WP groups, with geographical distance being the most important factor, followed by other environmental factors. Meanwhile, different microeukaryotic groups exhibited distinct western-eastern PO distribution patterns (Fig. 5), in response to distinct environmental variables, as revealed by the Spearman correlation analysis between microeukaryotic groups and environmental variables (Fig. S11). Similar grouping patterns of microeukaryotic communities were also discovered in the western PO, with geographical distance, temperature, and salinity identified being the primary drivers (21). Our study revealed that 150 out of 5,957 ZOTUs occurred in all samples. These ZOTUs were not only widely distributed but also well presented in all samples, as the majority of ZOTUs were either abundant or intermediate and were only rare in a few samples. Sun et al. (68) proposed that the intermediate ciliate group played an important role in sustaining the stability and functionality of the ciliate community by transitioning between the abundant and rare groups (68). As demonstrated by our research, the intermediate group may also play an essential role in maintaining microeukaryotic communities on a much larger spatial scale in the PO.

In the present study, dispersal limitation and heterogeneous selection were found to be the primary determinants of the community structure of microeukaryotes, resulting in a negative correlation between community similarity and geographic distance (the distance-decay pattern) (69). Martiny et al. (70) discovered that the limitation on microbial dispersal increases with geographic distance (70). Studies have demonstrated that dispersal limitation caused by geographical distance is a key factor in shaping the planktonic microeukaryote community in the ocean (10, 34, 41, 71–73). Simultaneously, environmental gradients have a substantial effect on microeukaryotic assembly and, in conjunction with the geographic scales, explain the balance between deterministic and stochastic processes (69). According to the results of our VPA analysis, 62.7% of the variation remained unexplained, which may be due to unmeasured environmental and ecological factors. Studies have indicated that species interactions, such as competition, parasitism, predation, symbiosis, and phycosphere among microeukaryotes, play an important role in community structure (6) and distribution patterns (4, 15, 21, 65).

### Conclusion

This study investigated the biodiversity and biogeography of planktonic microeukaryotes across the tropical PO. Alpha diversity indices of microeukaryotes decreased along the west-to-east PO transect, with water temperature being the most important driving factor. The microeukaryotic communities displayed a clear distance-decay pattern and were clustered into three discrete groups according to the sampling site locations: the western PO group, the central PO group, and the eastern PO group. Geographical distance was identified as the primary driver of community changes, highlighting the significance of geographical distance in shaping microeukaryotic communities, particularly on large spatial scales. The parasitic dinoflagellate lineage Syndiniales was found to be the most abundant (sequence proportion) and diverse (ZOTU richness) microeukaryotic group, and their wide distribution and close correlation with other microeukaryotic groups, including dinoflagellates, ciliates, diatoms, and so forth, suggest that parasitism may be an important living strategy of planktonic microeukaryotes in surface waters of the PO, whose functions in biogeochemical cycling require further investigation.

## SUPPLEMENTAL MATERIAL

Supplemental material spectrum.02424-23-s0001.docx Supplemental figures and tables.

ASM does not own the copyrights to Supplemental Material that may be linked to, or accessed through, an article. The authors have granted ASM a non-exclusive, world-wide license to publish the Supplemental Material files. Please contact the corresponding author directly for reuse.
