# Supplementary material for: Biogeographical and biodiversity patterns of planktonic microeukaryotes along the tropical western to eastern Pacific Ocean transect revealed by metabarcoding
Source: Microbiol Spectr. 2024 Mar 15;12(4):e02424-23. doi: 10.1128/spectrum.02424-23 (PMC10986530; doi:10.1128/spectrum.02424-23)
Supplement: Supplemental material — Supplemental figures and tables. [file spectrum.02424-23-s0001.docx]

**Fig. S1.** The UPGMA clustering of microeukaryotic communities. EP, the eastern Pacific Ocean group; CP, the central Pacific Ocean group; WP, the western Pacific Ocean group.


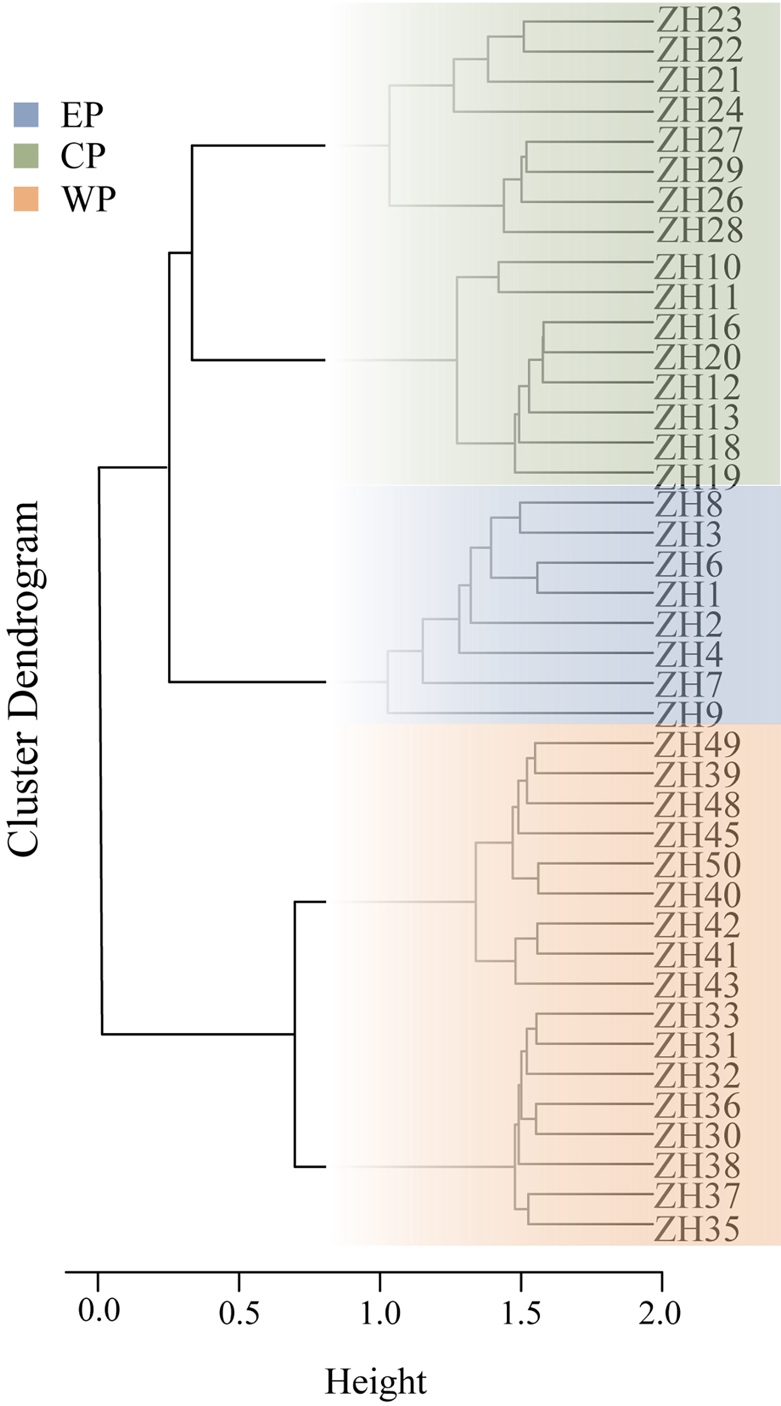


**Fig. S2.** Linear regression plots of alpha diversity indexes (ZOTU Richness, Shannon, and PD).


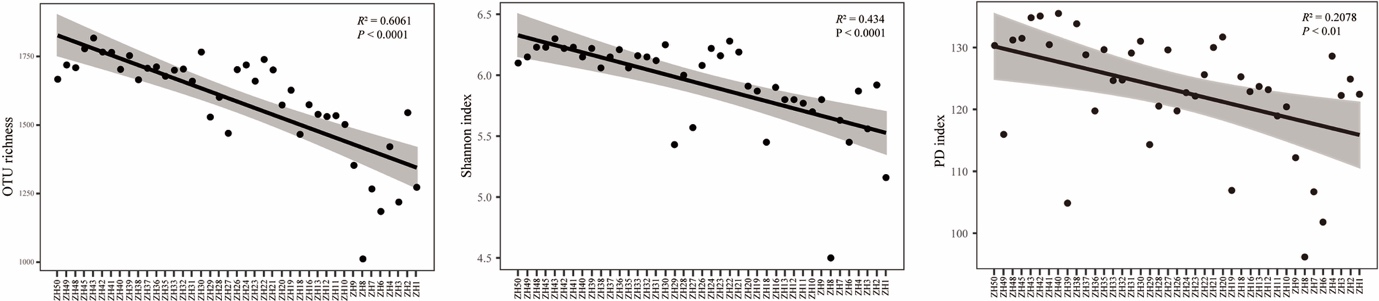


**Fig. S3.** Multiple linear regression (MLR) model analysis between the alpha diversity indices and the environmental parameters.


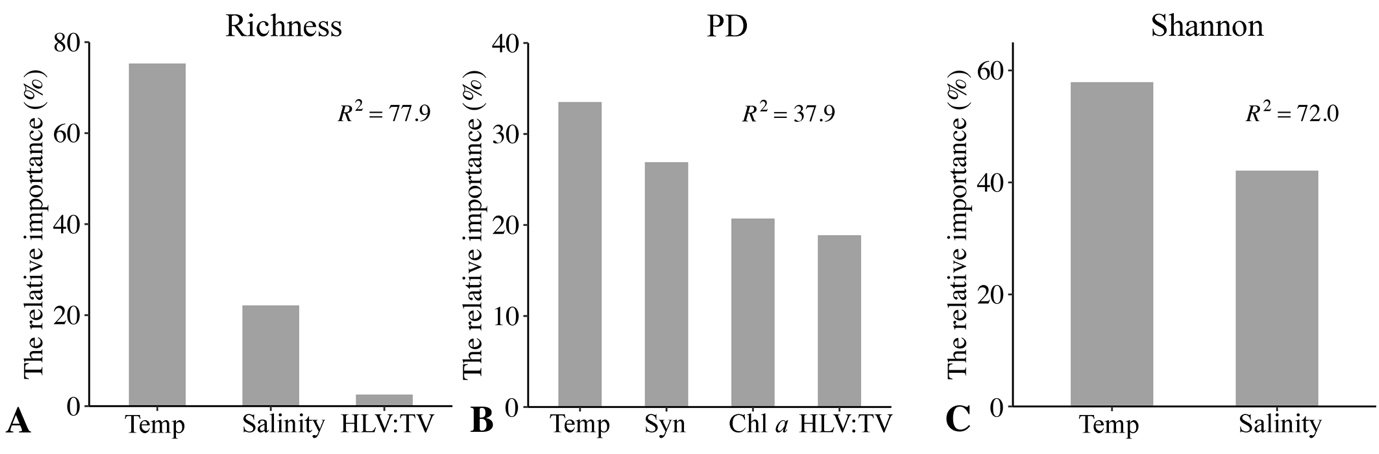


**Fig S4.** Composition of microeukaryotic communities for the pooled dataset at the supergroup level (A, read proportion; B, ZOTU richness proportion) and for individual samples at the phylum level (A, read proportion; B, ZOTU richness proportion).


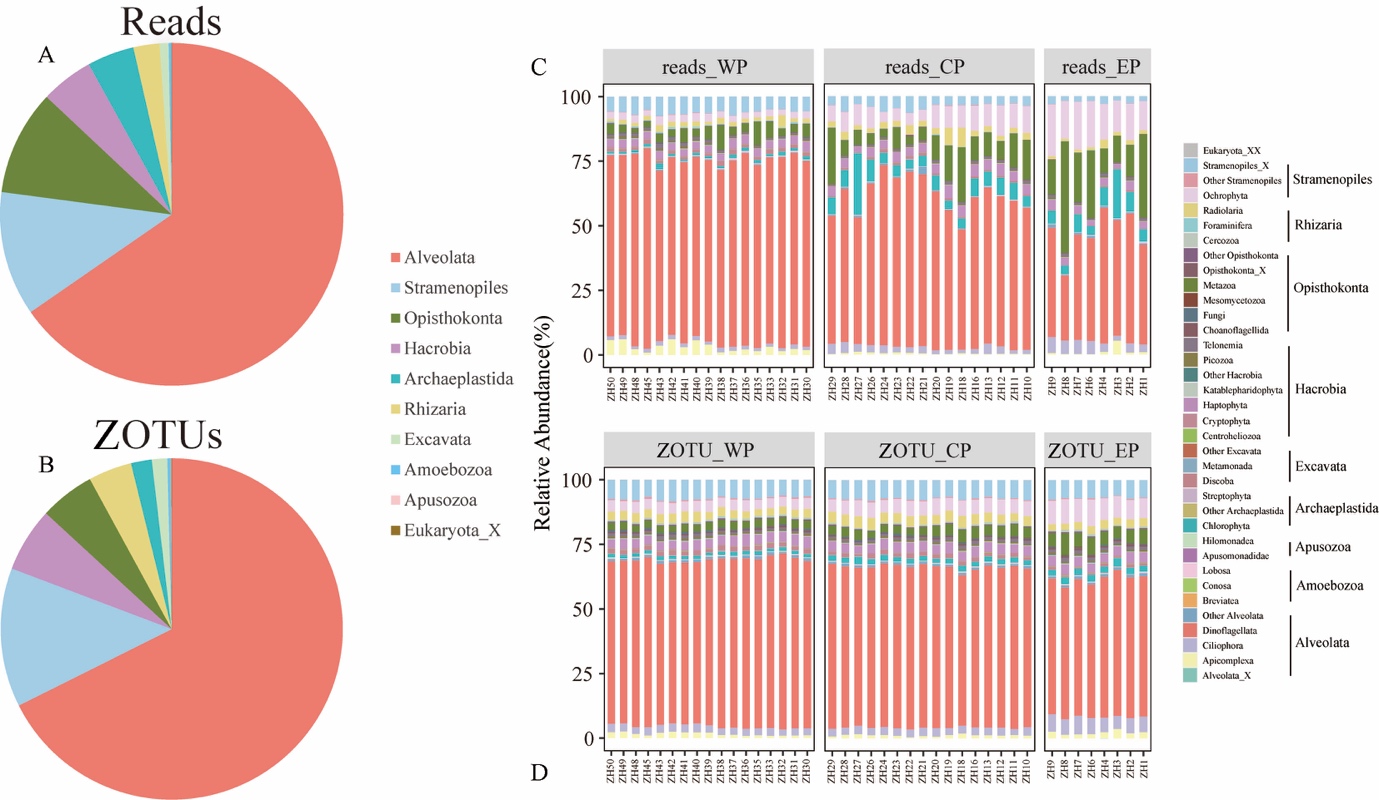


**Fig. S5.** Heatmap showing the distribution of ZOTUs that were found in all samples.


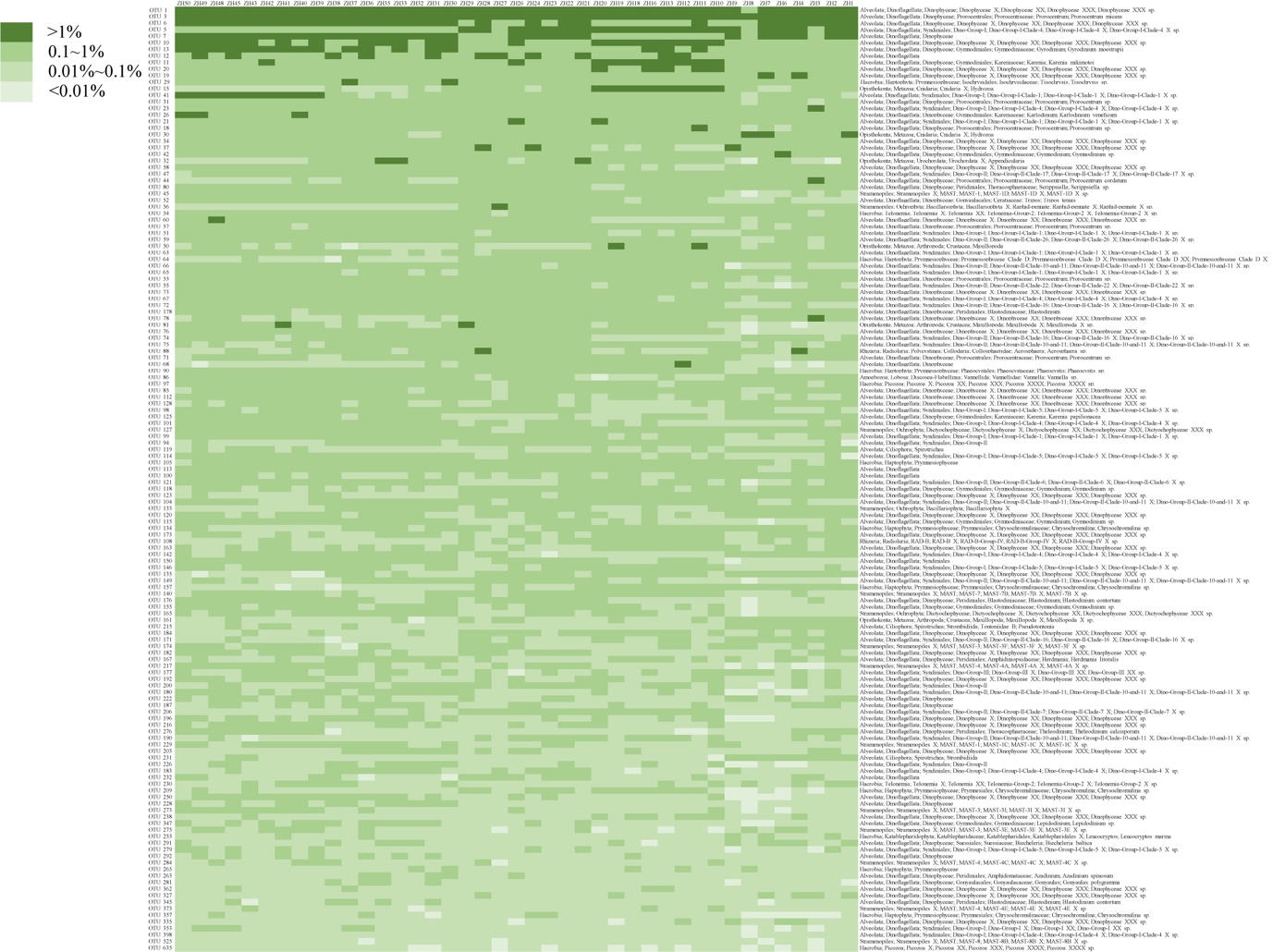


**Fig. S6.** Co-occurrence patterns of microeukaryotes.


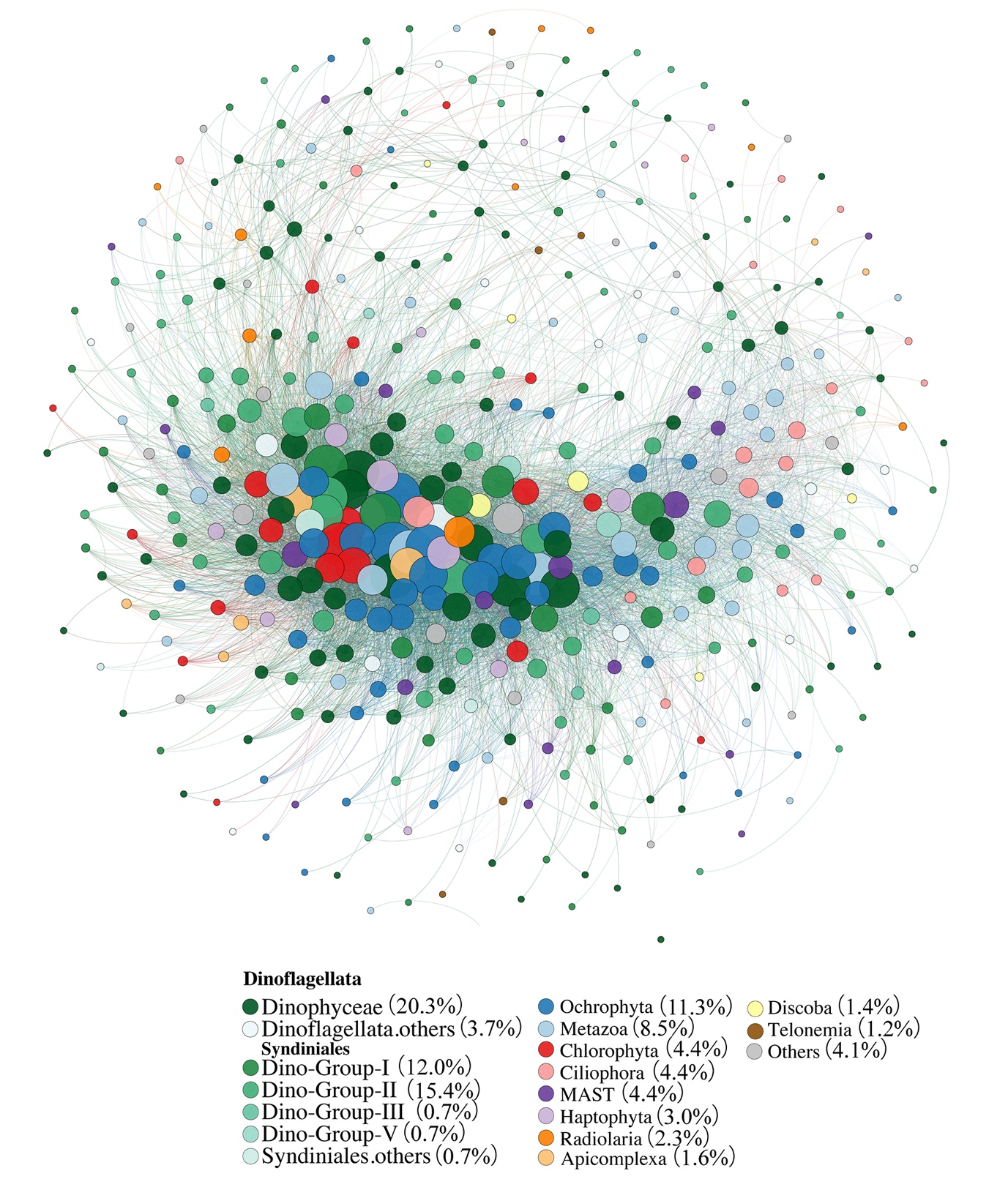


**Fig. S7.** Variations in sequence proportions and ZOTU richness of different functional groups in the three geographical groups. ND, not determined.


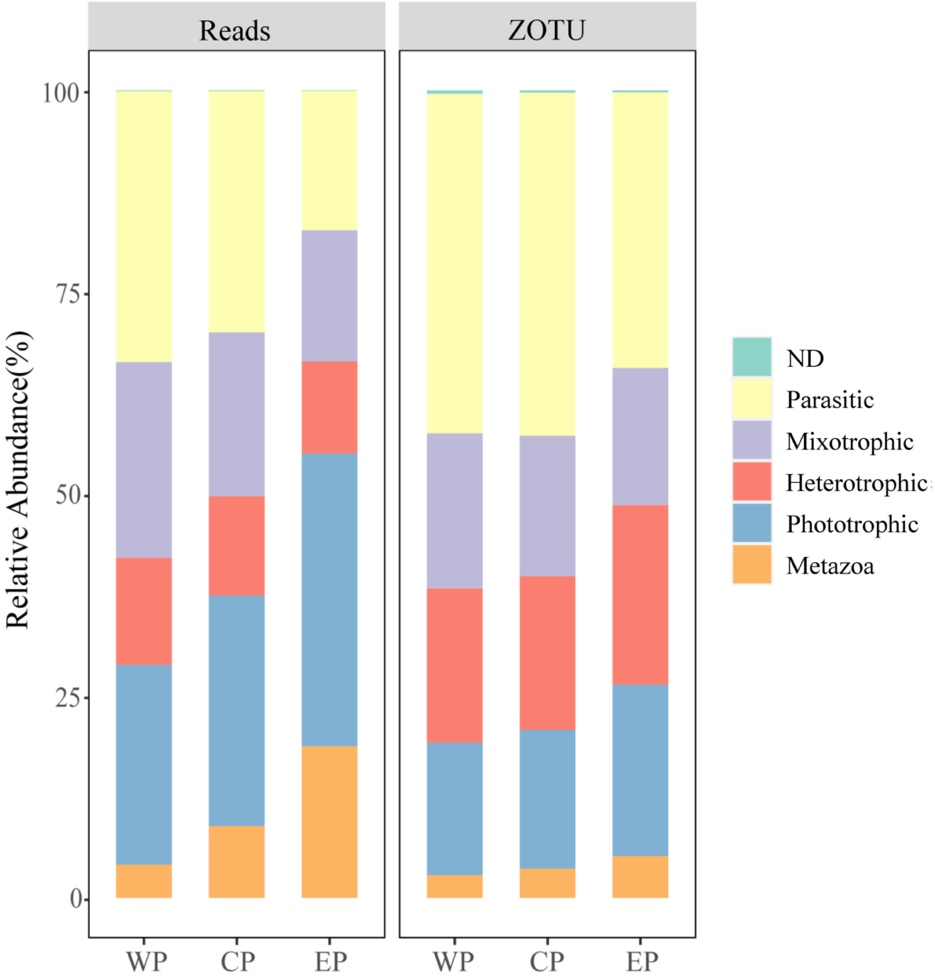


**Fig. S8.** Proportions of reads (upper) and ZOTUs (lower) of different tropical groups of microeukaryotes in each sample.


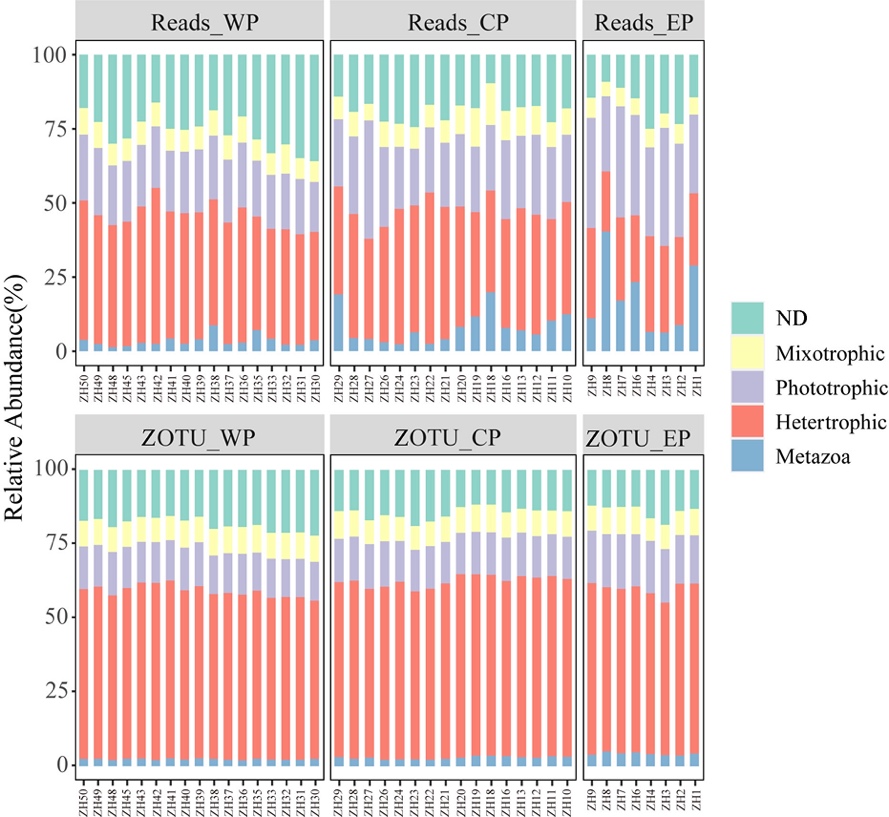


**Fig. S9.** Distance decay pattern of microeukaryotic communities in the Pacific Ocean.


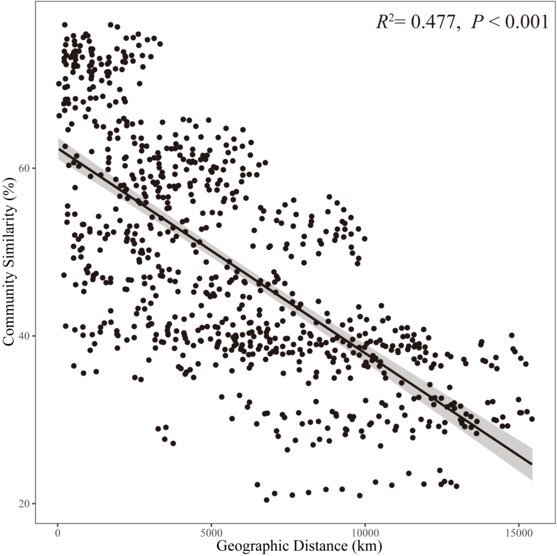


**Fig. S10.** The distribution of water temperature along the sampling transect.

**
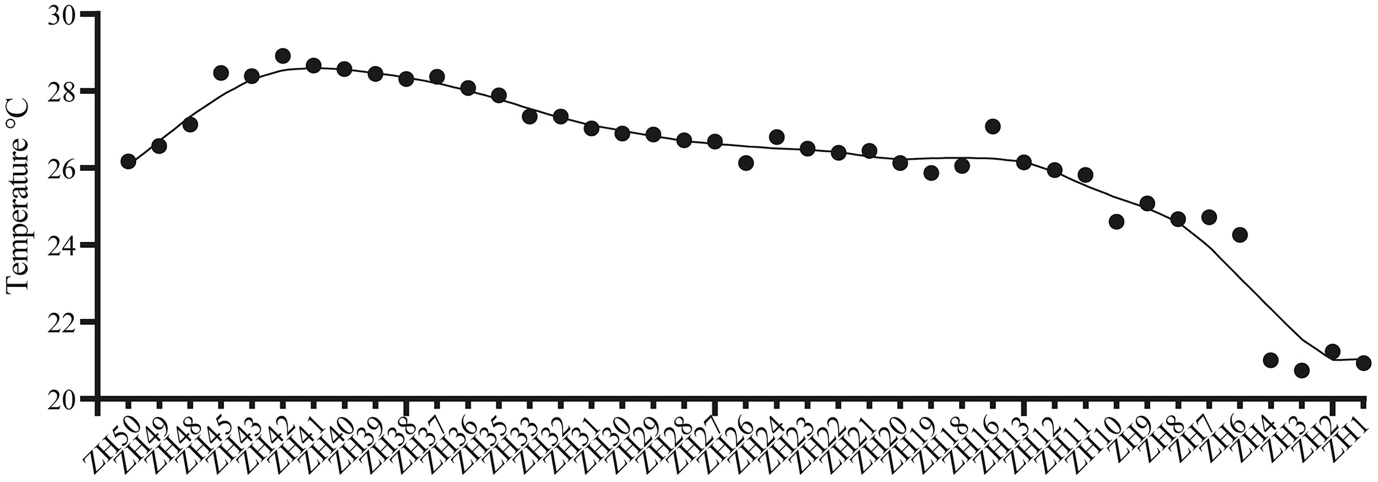
**

**Fig. S11.** Spearman’s correlations between relative abundance of major microeukaryotic groups at phylum level and environmental parameters. The correlation coefficients’ values are indicated according to the color bar.


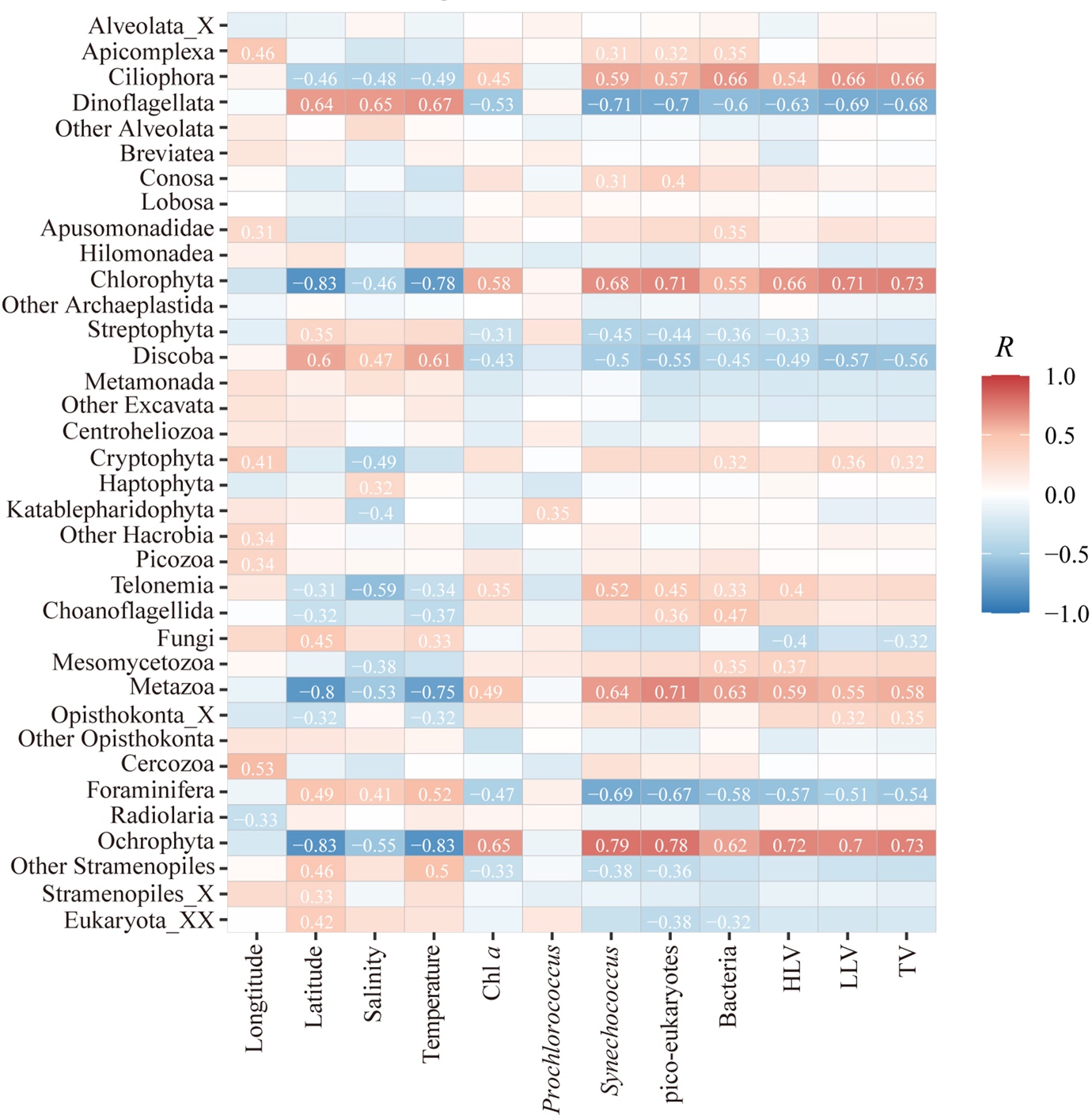


**Table S1.** Sample information and alpha diversity indices of each sample. *Subsampled at 11,783 reads.

| Station | Sample name | Lat.  (°N) | Long.  (°E) | Clean Reads | ZOTUs | ZOTUs* | Shannon* | PD* |
| --- | --- | --- | --- | --- | --- | --- | --- | --- |
| ZH50 | ZH50 | 25.75 | 127.67 | 29477 | 2385 | 1667 | 6.10 | 130.33 |
| ZH49 | ZH49 | 25.24 | 129.74 | 30346 | 2461 | 1719 | 6.15 | 115.97 |
| ZH48 | ZH48 | 24.15 | 132.28 | 11783 | 1709 | 1709 | 6.23 | 131.21 |
| ZH45 | ZH45 | 17.44 | 135.09 | 29496 | 2538 | 1778 | 6.23 | 131.48 |
| ZH43 | ZH43 | 17.32 | 135.53 | 28108 | 2515 | 1817 | 6.30 | 134.81 |
| ZH42 | ZH42 | 17.18 | 140.51 | 24821 | 2384 | 1766 | 6.22 | 135.08 |
| ZH41 | ZH41 | 16.54 | 145.28 | 32910 | 2673 | 1765 | 6.23 | 130.46 |
| ZH40 | ZH40 | 15.82 | 150.57 | 26014 | 2351 | 1703 | 6.15 | 135.50 |
| ZH39 | ZH39 | 15.14 | 155.61 | 15211 | 1943 | 1753 | 6.22 | 104.87 |
| ZH38 | ZH38 | 14.46 | 160.43 | 65360 | 3058 | 1665 | 6.06 | 133.83 |
| ZH37 | ZH37 | 13.72 | 165.77 | 67057 | 3082 | 1707 | 6.15 | 128.83 |
| ZH36 | ZH36 | 13.58 | 170.65 | 33320 | 2555 | 1712 | 6.21 | 119.76 |
| ZH35 | ZH35 | 13.50 | 175.88 | 29183 | 2355 | 1678 | 6.06 | 129.66 |
| ZH33 | ZH33 | 13.42 | -179.24 | 69647 | 3155 | 1700 | 6.16 | 124.67 |
| ZH32 | ZH32 | 13.33 | -173.93 | 48558 | 2811 | 1704 | 6.15 | 124.74 |
| ZH31 | ZH31 | 13.29 | -171.52 | 66414 | 3103 | 1660 | 6.12 | 129.11 |
| ZH30 | ZH30 | 13.24 | -168.76 | 56937 | 2992 | 1766 | 6.25 | 131.02 |
| ZH29 | ZH29 | 13.13 | -166.36 | 27972 | 2188 | 1529 | 5.43 | 114.32 |
| ZH28 | ZH28 | 13.16 | -163.37 | 46008 | 2756 | 1601 | 6.00 | 120.54 |
| ZH27 | ZH27 | 13.12 | -161.13 | 44507 | 2551 | 1470 | 5.57 | 129.62 |
| ZH26 | ZH26 | 13.07 | -158.02 | 25604 | 2292 | 1702 | 6.08 | 119.76 |
| ZH24 | ZH24 | 5.99 | -144.19 | 62446 | 3206 | 1719 | 6.22 | 122.72 |
| ZH23 | ZH23 | 6.10 | -142.05 | 53524 | 2949 | 1660 | 6.16 | 122.15 |
| ZH22 | ZH22 | 6.01 | -141.71 | 39097 | 2781 | 1739 | 6.28 | 125.61 |
| ZH21 | ZH21 | 6.00 | -139.78 | 55965 | 3092 | 1701 | 6.19 | 130.01 |
| ZH20 | ZH20 | 6.04 | -137.03 | 75046 | 3110 | 1573 | 5.91 | 131.69 |
| ZH19 | ZH19 | 6.00 | -134.73 | 30674 | 2400 | 1627 | 5.87 | 106.92 |
| ZH18 | ZH18 | 6.00 | -132.92 | 60290 | 2753 | 1466 | 5.45 | 125.27 |
| ZH16 | ZH16 | 6.00 | -127.75 | 66894 | 2951 | 1574 | 5.90 | 122.88 |
| ZH13 | ZH13 | 6.00 | -121.48 | 32540 | 2338 | 1539 | 5.80 | 123.70 |
| ZH12 | ZH12 | 6.65 | -119.49 | 73848 | 2996 | 1531 | 5.80 | 123.19 |
| ZH11 | ZH11 | 5.91 | -117.68 | 74792 | 3080 | 1534 | 5.77 | 118.95 |
| ZH10 | ZH10 | 5.13 | -114.94 | 53039 | 2753 | 1502 | 5.70 | 120.42 |
| ZH9 | ZH9 | 4.62 | -113.17 | 49767 | 2306 | 1353 | 5.80 | 112.21 |
| ZH8 | ZH8 | 3.81 | -110.38 | 52110 | 1934 | 1012 | 4.50 | 96.17 |
| ZH7 | ZH7 | 3.00 | -107.59 | 18984 | 1524 | 1267 | 5.63 | 106.70 |
| ZH6 | ZH6 | 2.09 | -104.55 | 51813 | 2128 | 1185 | 5.45 | 101.81 |
| ZH4 | ZH4 | 0.25 | -98.65 | 16898 | 1648 | 1421 | 5.87 | 128.61 |
| ZH3 | ZH3 | -0.78 | -95.57 | 17943 | 1457 | 1219 | 5.56 | 122.26 |
| ZH2 | ZH2 | -2.57 | -90.31 | 43672 | 2496 | 1545 | 5.92 | 124.90 |
| ZH1 | ZH1 | -2.81 | -88.26 | 53726 | 2289 | 1667 | 5.16 | 122.45 |

**Table S2.** Spearman correlation analysis between the alpha diversity indices and environmental factors. * indicated *p* < 0.05, ** indicated *p* < 0.01.

|  | ZOTU Richness | Shannon | PD |
| --- | --- | --- | --- |
| Salinity | .542^**^ | .534^**^ | .261 |
| Temperature | .803^**^ | .719^**^ | .523^**^ |
| Chl *a* | -.519^**^ | -.432^**^ | -.140 |
| *Prochlorococcus* | -.063 | -.078 | .040 |
| *Synechococcus* | -.637^**^ | -.518^**^ | -.385^*^ |
| PPTs | -.664^**^ | -.529^**^ | -.360^*^ |
| Bacteria | -.563^**^ | -.483^**^ | -.247 |
| HFV | -.609^**^ | -.513^**^ | -.361^*^ |
| LFV | -.608^**^ | -.528^**^ | -.261 |
| Total viruses | -.624^**^ | -.538^**^ | -.281 |

**Table S3.** Significant (*p* < 0.01) correlations identified by SparCC between Syndiniales ZOTUs and other ZOTUs.

| Syndiniales | Number of ZOTUs | Tax | Number of ZOTUs |
| --- | --- | --- | --- |
| Dino-Group-I | 52 | Dinophyceae | 65 |
|  |  | Ochrophyta | 41 |
|  |  | Metazoa | 31 |
|  |  | Chlorophyta | 17 |
|  |  | MAST | 15 |
|  |  | Ciliophora | 14 |
|  |  | Dinoflagellata.others | 13 |
|  |  | Haptophyta | 10 |
|  |  | Radiolaria | 6 |
|  |  | Apicomplexa | 6 |
|  |  | Discoba | 6 |
|  |  | Telonemia | 3 |
|  |  | Others | 13 |
|  |  | Dino-Group-II | 56 |
|  |  | Dino-Group-I | 38 |
|  |  | Dino-Group-V | 3 |
|  |  | Dino-Group-III | 3 |
|  |  | Syndiniales.others | 3 |
| Dino-Group-II | 67 | Dinophyceae | 64 |
|  |  | Ochrophyta | 39 |
|  |  | Metazoa | 31 |
|  |  | Chlorophyta | 17 |
|  |  | Ciliophora | 15 |
|  |  | MAST | 14 |
|  |  | Dinoflagellata.others | 11 |
|  |  | Haptophyta | 10 |
|  |  | Apicomplexa | 6 |
|  |  | Radiolaria | 5 |
|  |  | Discoba | 5 |
|  |  | Others | 14 |
|  |  | Dino-Group-II | 47 |
|  |  | Dino-Group-I | 33 |
|  |  | Dino-Group-V | 3 |
|  |  | Dino-Group-III | 3 |
|  |  | Syndiniales.others | 2 |
| Dino-Group-III | 3 | Dinophyceae | 15 |
|  |  | Ochrophyta | 14 |
|  |  | Metazoa | 7 |
|  |  | MAST | 5 |
|  |  | Haptophyta | 5 |
|  |  | Chlorophyta | 4 |
|  |  | Dinoflagellata.others | 3 |
|  |  | Apicomplexa | 1 |
|  |  | Discoba | 1 |
|  |  | Ciliophora | 1 |
|  |  | Radiolaria | 1 |
|  |  | Others | 1 |
|  |  | Dino-Group-II | 14 |
|  |  | Dino-Group-I | 8 |
|  |  | Dino-Group-V | 2 |
|  |  | Syndiniales.others | 1 |
| Dino-Group-V | 3 | Ochrophyta | 22 |
|  |  | Dinophyceae | 20 |
|  |  | Metazoa | 13 |
|  |  | Ciliophora | 5 |
|  |  | Dinoflagellata.others | 5 |
|  |  | MAST | 5 |
|  |  | Haptophyta | 5 |
|  |  | Chlorophyta | 5 |
|  |  | Discoba | 3 |
|  |  | Telonemia | 2 |
|  |  | Radiolaria | 1 |
|  |  | Apicomplexa | 1 |
|  |  | Others | 5 |
|  |  | Dino-Group-II | 19 |
|  |  | Dino-Group-I | 16 |
|  |  | Dino-Group-V | 1 |
|  |  | Dino-Group-III | 1 |
|  |  | Syndiniales.others | 1 |
| Syndiniales.others | 3 | Dinophyceae | 23 |
|  |  | Ochrophyta | 20 |
|  |  | Chlorophyta | 9 |
|  |  | Metazoa | 8 |
|  |  | MAST | 5 |
|  |  | Haptophyta | 5 |
|  |  | Dinoflagellata.others | 3 |
|  |  | Apicomplexa | 3 |
|  |  | Radiolaria | 3 |
|  |  | Discoba | 1 |
|  |  | Ciliophora | 1 |
|  |  | Others | 5 |
|  |  | Dino-Group-II | 18 |
|  |  | Dino-Group-I | 10 |
|  |  | Dino-Group-V | 1 |
|  |  | Dino-Group-III | 1 |
